# Discovery of a pyrano[2,3-*b*]pyridine derivative YX-2102 as a cannabinoid receptor 2 agonist for alleviating lung fibrosis

**DOI:** 10.1186/s12967-022-03773-1

**Published:** 2022-12-06

**Authors:** Tao Liu, Jing Gu, Yi Yuan, Qunfang Yang, Peng-Fei Zheng, Changyu Shan, Fangqin Wang, Hongwei Li, Xiang-Qun Xie, Xiao-Hong Chen, Qin Ouyang

**Affiliations:** 1 College of Pharmacy, Third Military Medical University, Chongqing, 400038 China; 2 Department of Pharmaceutical Sciences, School of Pharmacy, University of Pittsburgh, Pittsburgh, PA 15261 USA

**Keywords:** Cannabinoid 2 receptor, Pulmonary fibrosis, Virtual screening, Epithelial-to-mesenchymal transition, Nrf2-Smad7 pathway

## Abstract

**Background:**

Pharmacological modulation of cannabinoid 2 receptor (CB2R) is a promising therapeutic strategy for pulmonary fibrosis (PF). Thus, to develop CB2R selective ligands with new chemical space has attracted much research interests. This work aims to discover a novel CB2R agonist from an in-house library, and to evaluate its therapeutic effects on PF model, as well as to disclose the pharmacological mechanism.

**Methods:**

Virtual screening was used to identify the candidate ligand for CB2R from a newly established in-house library. Both in vivo experiments on PF rat model and in vitro experiments on cells were performed to investigate the therapeutic effects of the lead compound and underlying mechanism.

**Results:**

A “natural product-like” pyrano[2,3-*b*]pyridine derivative, YX-2102 was identified that bound to CB2R with high affinity. Intraperitoneal YX-2102 injections significantly ameliorated lung injury, inflammation and fibrosis in a rat model of PF induced by bleomycin (BLM). On one hand, YX-2102 inhibited inflammatory response at least partially through modulating macrophages polarization thereby exerting protective effects. Whereas, on the other hand, YX-2102 significantly upregulated CB2R expression in alveolar epithelial cells in vivo. Its pretreatment inhibited lung alveolar epithelial-to-mesenchymal transition (EMT) in vitro and PF model induced by transforming growth factor beta-1 (TGF-β1) via a CB2 receptor-dependent pathway. Further studies suggested that the Nrf2-Smad7 pathway might be involved in.

**Conclusion:**

These findings suggest that CB2R is a potential target for PF treatment and YX-2102 is a promising CB2R agonist with new chemical space.

**Supplementary Information:**

The online version contains supplementary material available at 10.1186/s12967-022-03773-1.

## Background

The endocannabinoid system is a promising therapeutic target in multiple fibrotic diseases. Endocannabinoids are internal lipid mediators that act on two known cannabinoid receptors (CBR), CB1R and CB2R [1]. The activation of CB2R can protect against fibrosis in various organs, including the kidney [2], heart [3], skin [4] and etc. Only a few studies have reported the role of CB2R in the development of pulmonary fibrosis (PF). CB2R-deficient mice are reported to experience earlier and augmented lung fibrosis [5]. A recent study found that CB2R activation via JWH-133 can alleviate BLM-induced PF in mice [6]. These findings indicate that targeting CB2R may serve as a therapeutic strategy for PF treatment.

Besides anti-fibrotic properties, CB2R has anti-inflammatory and anti-oxidative stress effects. For instance, CB2R activation can significantly decrease oxidative stress and downregulate the inflammatory cascade in various inflammatory diseases [7]. CB2R activation can protect skeletal muscle against ischemia-reperfusion injury by ameliorating oxidative damage [8]. Moreover, genetic deletion of CB2R can exacerbate acute inflammatory response and neutrophil recruitment [9]. Meanwhile, numerous studies have shown that excessive oxidative stress and sustained inflammatory response in the lung can aggravate the fibrotic process [10]. Therefore, the exact role of CB2R and the cellular pathways involved downstream of the CB2R in PF progression should be examined.

As a result, scientists have continuously developed various CB2R selective ligands, including selective agonists (tetrahydromagnolol [11] and JWH-133 [5, 6]) and inverse agonists (AM630 [12], Sch225336 [13], SR144528 [14] and sulfonamides derivatives [15]). The discovery of new scaffold CB2R ligands with better activity and controllable toxicity for potential therapeutic use remains attractive to researchers [16]. Natural products are ideal compound sources for seeking new drug leads, due to their structural diversities and complexities [17]. A natural product, Δ9-tetrahydrocannabinol (Δ9-THC) paved the way for understanding the functions of CBRs and the endocannobinoid system. Beside Δ9-THC directly separated from natural source, an array of its synthetic analogues was developed as CBR ligands. However, the source of Δ9-THC is limited, and the synthetic approach of its analogues is hampered by their structural complexity, largely limiting their further utilization and development.

Alternately, organic synthetic methodology studies have provided strategies to rapidly build structural diverse, complex chiral molecules with scaffolds that are frequently found in bioactive molecules [18], including oxazoles, indoles, bridged carbocycles and spiro skeletons, etc. These “natural product-like” compounds incorporate the advantages of natural products such as diversity and complexity, along with the benefits of synthetic pharmaceutical agents such as accessibility. Although researches have indicated that the new compounds might have good bioactive potentials [19], to further develop them as therapeutic agent’s remains unrealized. Therefore, there is still huge space in utilizing them for drug lead screening.

**Fig. 1:**
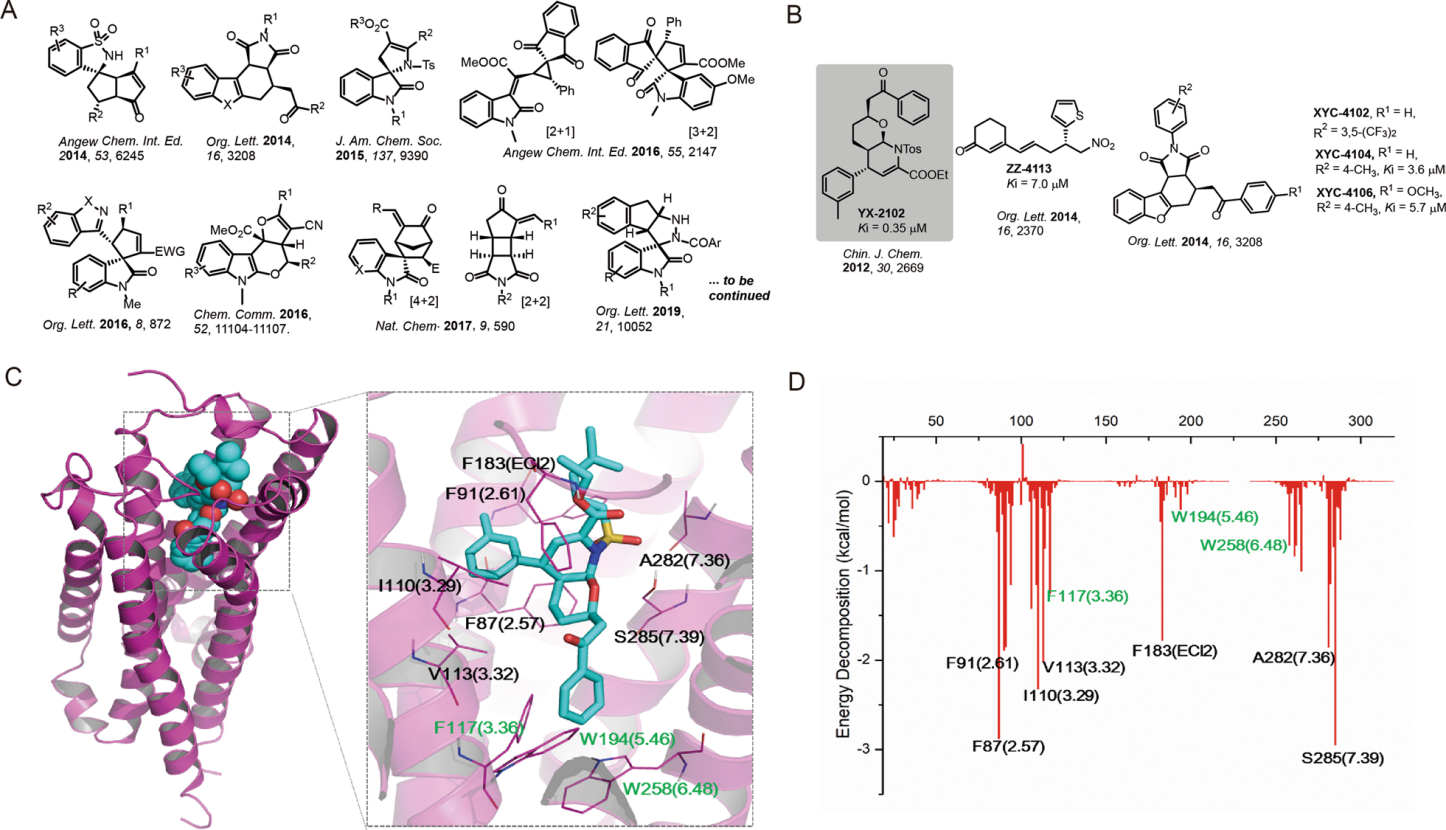
The discovery of the hit compound YX-2102. A Representative scaffolds collected from the natural product-like library. B Structures of YX-2102, XYC-4104, ZZ-4113, and XYC-4106 and their binding affinity (*Ki*) to CB2R. C Representative MD structure and the binding interaction of YX-2102 with CB2R (blue cartoon and brownish sticks). D Binding free energy decomposition of YX-2102/CB2 system

This study developed an in-house library covering 1600 chemicals obtained from various organocatalytic methodology studies reported by our group during 2012–2020 (representative structures in Fig. 1 A). The library was screened to identify new CB2R ligands. Virtual screening was first performed via molecular docking and dynamic simulation based on the reported CB2 X-ray structure since computational approaches have successfully been applied in CB2R ligand predictions [20]. The synthetic YX-2102, a pyrano[2,3-*b*]pyridine derivative was identified as a novel CB2R agonist. The potential therapeutic effects of YX-2102 on PF was then systematically investigated both in vivo and in vitro, as well as the underlying mechanisms. YX-2102 ameliorated lung fibrosis by suppressing EMT in a CB2R dependent manner via regulation of the Nrf2/Smad7 pathway, indicating that CB2R is a potential therapeutic target for PF treatment.

## Methods

### Reagents

Dulbecco’s modified eagle medium (DMEM) and fetal bovine serum (FBS) were purchased from Hyclone (UT, USA). Penicillin and streptomycin were purchased from Beyotime (Jiangsu, China). Human recombinant TGF-β1 was provided from Peprotech (Rocky Hill, NJ). Bleomycin sulfate was supplied by Nippon Kayaku Co., LTD. (Japan). Information including source, catalog, dilution ratio, and storage conditions of primary/secondary antibodies are presented in Additional file 1: Table S1, and the structural information and source of the active compounds are listed in Additional file 1: Table S2.

### Experimental animals and treatments

Male Sprague-Dawley rats (6–8 weeks old, weight: 180–200 g) were obtained from the Laboratory Animal Center of the Army Medical University (Chongqing, China). The Institutional Animal Care and Use Committee of the Army Medical University approved all animal experiments. The PF model was established as previously described [21]. Briefly, rats were administered with a single intratracheal instillation of 5 mg/kg BLM (in saline). The sham-operated rats underwent the same procedure, except for intratracheal instillation of normal saline instead of 5 mg/kg BLM. The rats were randomly divided into 4 groups (6 animals each): (1) Sham group (sham-operated rats received vehicle only), (2) YX-2102 group (sham-operated rats treated with YX-2102 alone), (3) BLM group (BLM-induced rats received the vehicle), and (4) BLM + YX-2102 group (BLM-induced rats treated with YX-2102). The vehicle was composed of a 0.9% saline: dimethyl sulfoxide (DMSO): Tween-80 (18:1:1). The YX-2102 was dissolved in this vehicle. The rats were intraperitoneally injected with 25 mg/kg YX-2102 daily or an equal volume of vehicle. The dose administered were selected based on the results of the preliminary experiments (Additional file 1: Fig. S1). All rats were sacrificed via cervical dislocation on day 7 (to observe the acute inflammatory responses) or day 21 (to observe the pulmonary fibrosis). Blood samples were then collected via cardiac puncture, and the serum was stored at − 80 °C for further experimentation. The bronchoalveolar lavage fluid (BALF) was collected, then the whole lung lobes were removed for histopathological and immunohistochemical (IHC) analyses (left lobes) or molecular and biochemical analyses (right lobes). All experiments followed relevant guidelines for the care and use of animals.

### Cell culture and treatments

Human alveolar epithelial adenocarcinoma cell line A549 (Cell Bank of the Chinese Academy of Sciences) and rat alveolar type II cell line RLE-6TN (American Type Culture Collection) were cultured in DMEM containing 10% FBS at 37 °C in a 5% CO_2_ atmosphere. All chemical compounds were dissolved in DMSO to a 10 mg/mL stock concentration, then stored at −20 °C. The cells were serum-starved for 18 h, then preincubated with the chemical compounds (YX-2102, JWH-133 or XL-002) at indicated concentrations for 2 h, and subsequently incubated with TGF-β1 (5 ng/mL) for 24 or 48 h. Total RNA was extracted and subjected to quantitative Real-Time PCR analysis. Proteins were extracted from cell lysates and were subjected to western blotting (WB) analysis. All measurements were performed at least 3 replicates.

### Histology, immunohistochemistry (IHC) and immunofluorescence (IF) assays

To visualize the complete tissue architecture and collagen deposition, hematoxylin and eosin (H&E) and Masson’s trichrome staining were processed according to a reported literature [22–24]. The alveolitis and fibrosis score was evaluated based on at least 10 randomly selected fields under microscope, the severity of alveolitis and fibrosis was semi-quantified as described by Szapiel et al. [25] and Ashcroft et al. [26]. Immunohistochemical staining and immunofluorescence experiments of lung tissue sections or cultured cells were performed following established procedures [22–24].

### Quantitative RT-PCR (qRT-PCR)

The qRT-PCR experiments were performed following the procedures reported previously [24]. The specific primer sequences for qRT-PCR are shown in the Additional file 1: Table S3.

### Enzyme-linked immunosorbent assays (ELISA)

ELISA was used to assess the levels of serum TGF-β1 via the ELISA kits (EK0514, Boster Bioengineering Institute, China), following the manufacturer’s instructions.

### Western blotting analysis

Tissues or cells were lysed using RIPA buffer (Beyotime, Jiangsu, China) on ice for 20 min, then quantified using a BCA assay kit (Sangon Biotech, China). The sodium dodecyl sulfate-polyacrylamide gel electrophoresis (SDS-PAGE) was used to separate proteins, then transferred to polyvinylidene fluoride (PVDF) membranes (Millipore Corp, USA) via electrophoresis (Bio-Rad, USA). The membranes were blocked with 5% non-fat milk in PBS with 0.05% Tween-20, then incubated with respective primary antibodies at 4 °C overnight. The protein bands were then stained with horseradish peroxidase (HRP)-conjugated secondary antibodies, and the immunoreactive proteins were visualized using the enhanced chemiluminescence reagent (ECL; Thermo Fisher, USA). Images were obtained using ChemiDoc™ Touch Imaging System (Bio-Rad, USA) and analyzed using Image Lab packages (Bio-Rad).

### RNA interference

The siRNA targeting human CB2R (sc-41,586, Santa Cruz, USA) and control siRNA were transferred into A549 cells at 10 µM concentration using lipofectamine 2000 (Invitrogen) following the manufacturer’s instructions. After 6 h, the medium was replaced with a complete medium and cells were cultured for another 48 h, then collected for further experiments.

### Hydroxyproline assay

Lung hydroxyproline (Hyp) content is a tissue marker for collagen. Herein, Hydroxyproline Analysis Kit (Jiancheng, China) was used to measure lung Hyp content, following the manufacturer’s instructions. The content was expressed as micrograms Hyp per milligram wet weight (µg/mg).

### Micro-computed tomography (CT)

The rats were anaesthetized using isoflurane and the lungs were imaged using a Quantum FX Micro-CT scanner (PerkinElmer Inc., MA) as previously reported [27]. The Mimics 17.0 (Materialise Software, Belgium) was used to convert the acquired data into 3D models to show airways, lung lobes and fibrosis.

### Bronchoalveolar lavage fluid (BALF)

The BALF was obtained by cannulating the trachea and infusing with 10 mL ice-cold 0.9% saline thrice. About 60–80% of fractions were recovered. The BALF was centrifuged (2000 rpm, 10 min, 4˚C), and the obtained supernatant was stored at − 80 °C for further experimentation. The cell sediments were re-suspended and stained with a modified Wright–Giemsa solution (Jiancheng, Nanjing, China). Differential cell counts were determined using 400 lung inflammatory cells.

### Molecular docking

The crystal structure of CB2R was obtained from RCSB Protein Data Bank (ID: 5ZTY) [20]. Before docking, the protein structure was prepared as reported [28, 29], using SYBYL-X 2.0 software and PDB2PQR Server [30]. The binding pocket was generated based on the ligand 9JU with default settings. The Surflex-Dock in SYBYL-X 2.0 software (SFXC mode) was used for the docking of the compound library CB2R. The following parameters were set on: pre-dock minimization/post-dock minimization/consider ring flexibility/molecule fragmentation/soft grid treatment. Obtained docked complexes with the highest scores (top 5) were subjected for molecular dynamic simulation (MD). The binding interactions between the ligand and CB2R were characterized using LigPlot+ [31].

### MD simulation, trajectory analysis and calculation of binding free energies

The structure preparation, MD simulation, the trajectory analysis and binding free energies calculation were performed using different modules of AMBER14. All detailed procedures and parameter settings followed the previous articles reported by our group [28, 29].

### CB2R binding assay

The affinities of compounds with human CB2 receptors were determined using a radioligand binding assay as previously described [25] [using [3 H](−)-cis-3-[2-hydroxy-4-(1,1-dimethylheptyl)phenyl]-trans-4-(3-hydroxypropyl)cyclohexanol (CP 55,940) as CB receptor radioligand].

### Statistical analysis

The data are shown as the mean ± SEM, and significance was established when P < 0.05. One-way analysis of variance (ANOVA) followed by the LSD or Dunnett’s test was used for pairwise comparisons.

## Results

### Screening and identifying YX-2102 as the potential ligand for CB2R

The in-house chemical library was *in silico-*screened to identify the potential ligand for CB2R. Molecular docking was conducted between CB2R and the 1600 + chemicals collected in the library (representative scaffolds are listed in Fig. 1A). The interaction was evaluated by analyzing docking scores and binding poses. The YX-2102, XYC-4102, XYC-4104, ZZ-4113, and XYC-4106 were predicted as the promising candidates, with docking scores higher than 8.0 (Fig. 1B). The binding affinities of the compounds were then evaluated. All the compounds interacted with CB2R with moderate affinity except for XYC-4102 that showed no binding. The *Ki* values of ZZ-4113, XYC-4104, XYC-4106 were 7.0, 3.6 and 5.7 µM, respectively. The binding affinity of YX-2102 with CB2R was much higher *(Ki*, 0.35 µM) than that of other compounds, suggesting that YX-2102, a pyrano[2,3-*b*]pyridine derivative with continuous chiral centers, was the most promising candidate for CB2R binding (Additional file 1: Fig. S2).

### Binding interaction analysis of YX-2102 with CB2R

**Fig. 2:**
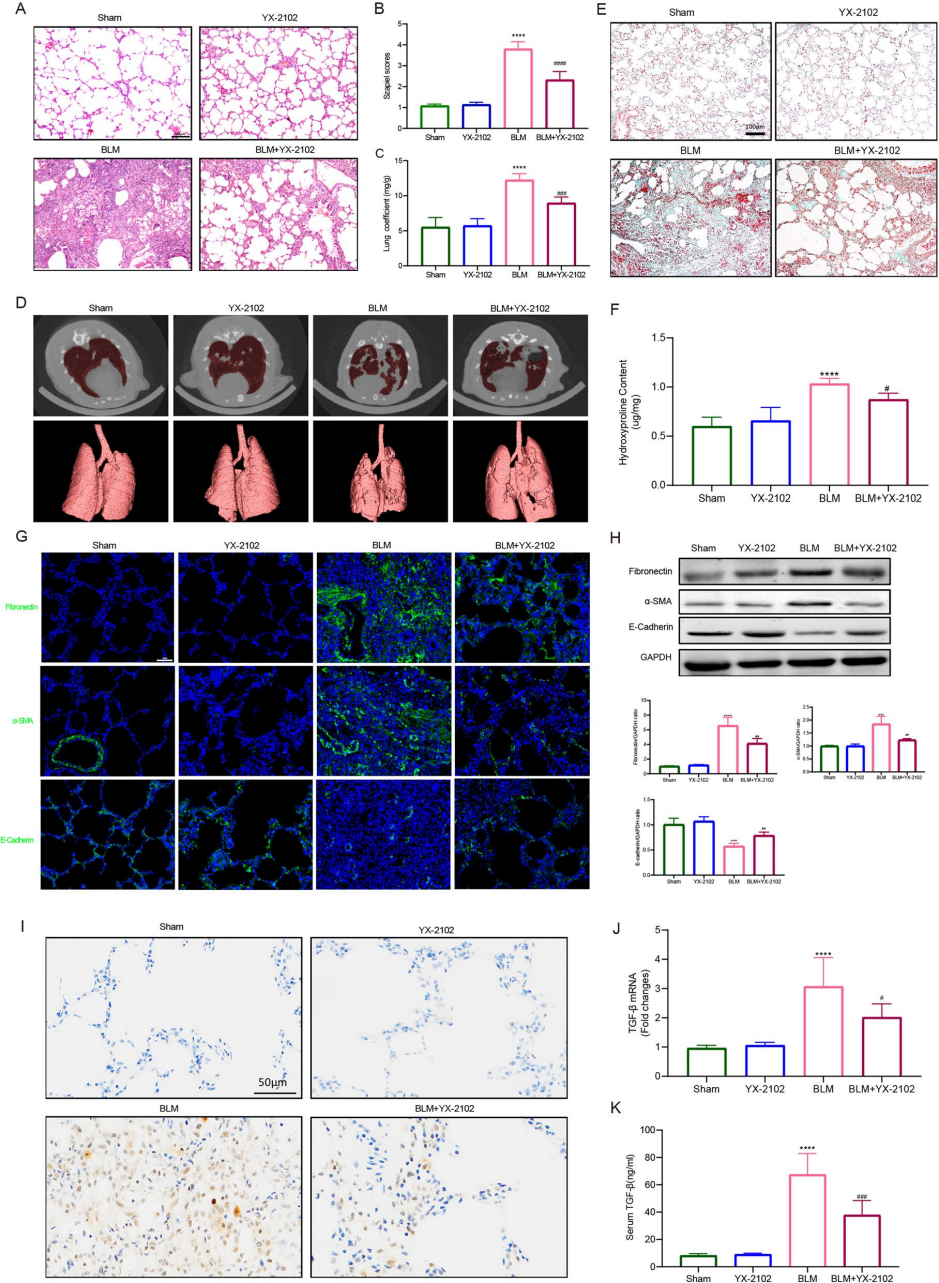
YX-2102 can protect against BLM-induced PF in vivo. Rats were intratracheally treated with 5 mg/kg BLM once at day 0, followed by YX-2102 or vehicle treatment via intraperitoneal injection at 25 mg/kg daily. **A** H&E staining of representative lung sections of the sham, YX-2102, BLM and BLM + YX-2102 groups at day 21. **B** Quantification of pulmonary fibrosis using the Szapiel score. **C** Lung coefficient index. **D** Micro-CT scan images of rats (in vivo) at 21 days after exposure. **E** Representative images of Masson’s trichrome staining of rat lungs at day 21. **F** The Hyp level of the pulmonary tissues. **G** Representative immunofluorescence staining images showing the expression of fibronectin, α-SMA and E-cadherin in rat lungs. **H** Western blotting showing the expression of fibronectin, α-SMA and E-cadherin in rat lungs. Histograms of protein ratios were normalized to a marker protein—the cellular skeletal glyceraldehyde-3-phosphate dehydrogenase (GAPDH, n = 3). **I**–**K** TGF-β1, the major pro-fibrotic factor, as identified by immunohistochemical assay (**I**) and quantitative RT-PCR (**J**) in lung tissues and ELISA (**K**) in rat serum at day 21. Scale bar is indicated in the figure. Data were expressed as mean ± SEM, n = 5, ^****^P < 0.0001 versus Sham groups, ^#^P < 0.05, ^###^P < 0.001, ^####^<0.0001 versus BLM group

The structure of docked YX-2102/CB2R complex were subjected to 150 ns molecular dynamics simulations (performed in Amber 14) to provide insights into their binding mechanisms. The RMSD was monitored until stabilization of the binding pose. CB2R and YX-2102 reached equilibrium at about 40 ns. The MM/GBSA free energy was also calculated to analyze the interactions. The total binding free energy of the YX-2102/CB2R system was − 69.9 ± 4.2 kcal/mol (Additional file 1: Table S4). The results showed that the ΔEvdw (van der Waals, VDW) significantly promoted the binding force (− 76.4 ± 3.1 kcal/mol), suggesting that the interactions were primarily mediated by VDW force. The energy decomposition in the repaglinide-M^pro^ system was also calculated (Fig. 1 C). The results showed that F87^2.57^ and F91^2.61^ in TM2, I110^3.29^, V113^3.32^ and F117^3.36^ in TM3, F183 in ECL2, and A282^7.36^ and S285^7.39^ in TM7 contributed considerable energies (lower than − 1.5 kcal/mol), consistent with the key residues for the CB2R/ligand binding [20, 32]. A previous study showed that the binding interactions and energy contributions from W258^6.48^, W194^5.46^, and F117^3.36^ are critical for antagonist (AM10257) compared with agonist (WIN 55,212), and can be used to distinguish agonist and antagonist [33]. The benzyl group of YX-2102 was inserted into the binding pocket (Fig. 2C). Herein, the binding interactions of W258^6.48^, W194^5.46^, and F117^3.36^ were relatively weak, especially for W194^5.46^, suggesting that YX-2102 might be an agonist.

### YX-2102 alleviates bleomycin-induced pulmonary fibrosis in vivo

This study established a bleomycin-induced PF in rats to explore whether YX-2102 has anti-fibrotic effects on PF. YX-2102 was safe and well-tolerated with no serious adverse effects. For instance, YX-2102 alleviated the significant hyperaemia, congestion and edema in the lung morphology of the BLM group (Additional file 1: Fig. S3). Body weight change is an important indicator of disease severity in bleomycin-induced lung injury [34]. In general, bleomycin instillation led to body weight loss with subsequent increase in lung coefficients. With YX-2102 administration, the rats showed a higher body weight increase from day 7 compared with the “untreated” bleomycin group. (Additional file 1: Fig. S4), suggesting some remedial effect of YX-2102. The H&E staining showed that the rat lungs in the BLM group had markedly thickened alveolar walls, collapsed alveoli, increased interstitial tissue, as well as obvious inflammatory changes compared with the sham group. However, YX-2102 significantly relieved the severity of BLM-induced lung injury (Fig. 2A). Semi-quantitative analysis of the BLM-induced histological changes using the Szapiel score showed that YX-2102 treatment resulted in less severe fibrotic lesions (Fig. 2B). Furthermore, BLM stimulation significantly increased lung coefficients (lung wet weight/body weight). However, YX-2102 significantly decreased the lung coefficients (Fig. 2C). Lung Micro-CT scan also demonstrated that BLM induced lung fibrosis in the rats, while YX-2102 attenuated lung fibrosis (Fig. 2D and Additional files 2: Video S1, Additional file 3: Video S2, Additional file 4: Video S3, Additional file 5: Video S4). Moreover, YX-2102 significantly reduced the excessive deposition of extracellular matrix (ECM), as indicated by the Masson’s trichrome-positive areas (Fig. 2E). The Hyp contents in rat lung tissue were measured to evaluate the extent of PF since Hyp is the one of the main components in extracellular collagen. Rats in BLM group had higher Hyp level than the sham group. However, YX-2102 treatment significantly decreased the elevations in Hyp level (Fig. 2F), suggesting the protective role of YX-2102 in counteracting ECM accumulation.

This study then analyzed the expression of alpha-smooth muscle actin (α-SMA), fibronectin and E-cadherin through immunostaining, WB and qRT-PCR to assess whether YX-2102 inhibits experimental fibrosis. The BLM significantly increased the fibronectin and α-SMA expression in lung tissues while decreased E-cadherin expression. In contrast, YX-2102 significantly decreased fibronectin and α-SMA while increasing E-cadherin levels (Fig. 2G, H). Furthermore, the level of the major pro-fibrotic factor TGF-β1 was substantially decreased in the serum and lung tissues of the YX-2102-treated group (Fig. 2I, J, K). Overall, these findings indicate that YX-2102 can attenuate BLM-induced PF in vivo.

### YX-2102 alleviates bleomycin-induced pulmonary inflammatory responses

**Fig. 3:**
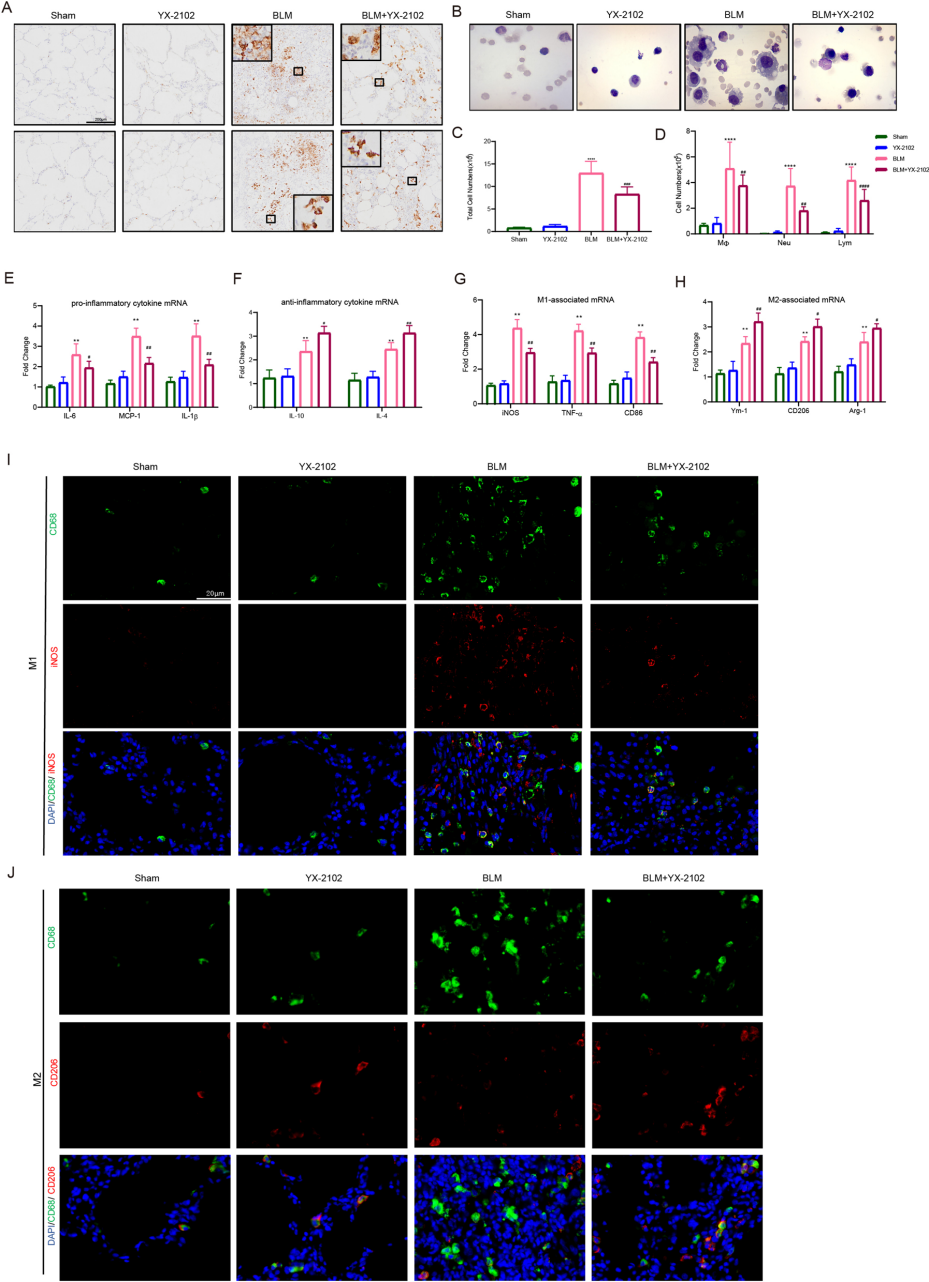
YX-2102 inhibits BLM-induced pulmonary inflammation in vivo. Rats were intratracheally treated with 5 mg/kg BLM once at day 0, followed by YX-2102 or vehicle treatment via intraperitoneal injection at 25 mg/kg daily. The rats were sacrificed at day 7 and sampled. **A** Immunohistochemical staining of lung tissues using anti-MPO or CD68 antibody. **B** Representative images of cells in BALF after Wright-Giemsa staining. **C** The cell count of BALF using a hemocytometer and **D** the number of macrophage (Mφ), lymphocytes (Lym) and neutrophils (Neu). **E**, **F** The mRNA expression of IL-6, MCP-1, IL-1β (**E**) and IL-10, IL-4 (**F**). **G**, **H** The mRNA level of TNF-α, iNOS, CD86 (**G**) and Ym-1, Arg-1, CD206 (). **I**, **J** Double immunofluorescence staining of CD68 (red)/iNOS (green) (**I**) and CD68 (red)/CD206 (green) in rat lungs. Nuclei were stained with DAPI (blue). The merged signal (yellow) indicated M1 or M2 macrophages, respectively. Data were expressed as mean ± SEM, n = 5, ^**^P < 0.01, ^***^P < 0.001 versus Sham groups. ^#^P < 0.05, ^##^P < 0.01, ^###^P < 0.001, ^####^P < 0.0001 versus BLM group

Inflammation is a causative factor in PF pathogenesis [35]. CB2R and its potential anti-inflammatory effect have been widely studied [36]. This study evaluated the effects of YX-2102 on the early inflammatory stage (day 7) of BLM-induced PF. First, IHC for myeloperoxidase (MPO) and CD68 was used to determine neutrophil and macrophage levels in the lung. The results indicated that neutrophil and macrophage infiltration increased in the BLM group. However, the YX-2102 treatment significantly attenuated the increase (Fig. 3A). Similarly, BLM significantly increased the total macrophage, neutrophil and lymphocyte cell numbers in bronchoalveolar lavage fluid (BALF) at day 7 compared with the sham-operated rats. However, the YX-2102 treatment significantly decreased the levels (Fig. 3B, C, D). The mRNA expression levels of both pro-inflammatory cytokines including IL-6, monocyte chemoattractant protein-1 (MCP-1) and IL-1β, as well as anti-inflammatory cytokines including IL-10 and IL-4 were detected in rat lung tissue. The BLM group had higher levels of IL-6, MCP-1 and IL-1β than the sham group (Fig. 3E). However, the YX-2102 treatment significantly decreased the expression of the pro-inflammatory cytokines. Moreover, the expression levels of the IL-10 and IL-4 cytokines [37, 38] were promoted in both BLM and BLM + YX-2102 groups. Meanwhile, the expression of the anti-inflammatory cytokines was obviously augmented in the YX-2102 treated group compared with the BLM group (Fig. 3F).

Furthermore, the expression of genes associated with M1 [inducible nitric oxide synthase (iNOS), TNF-α and CD86] or M2 [Ym-1, arginase-1 (Arg-1) and CD206] macrophage polarization was determined in the lung tissue using qRT-PCR to examine whether YX-2102 affects the polarization profile of M1 and M2 macrophage subset after BLM-induced lung injury. The expression of M1 and M2 markers significantly increased in rat lungs at seven days after BLM treatment. However, YX-2102 treatment significantly reduced the levels of M1-associated mRNA while it increased M2-associated mRNA levels (Fig. 3G, H).

To identify the type of accumulated macrophages in the fibrotic lung, immunofluorescence staining of CD68 (a macrophage marker) together with iNOS (to indicate M1-like macrophages) (Fig. 3I), or with CD206 (to indicate M2-like macrophages) (Fig. 3J) in lung tissues was performed. The co-expression of CD68 and iNOS was detected in infiltrated macrophages of the BLM group, consistent with the qPCR results. However, YX-2102 significantly decreased the proportion of M1-like macrophages. In contrast, the CD68 and CD206 co-expression were not detected in the BLM group. However, YX-2102 treatment significantly increased the numbers of M2-like macrophages. The above results implied that YX-2102 can inhibit BLM-induced pulmonary inflammation by inhibiting pro-inflammatory M1 macrophage polarization [39, 40], as well as increasing the anti-inflammatory M2 macrophages at early stage of inflammation.

### YX-2102 inhibits TGF-β1-induced EMT via a CB2R-dependent pathway

**Fig. 4:**
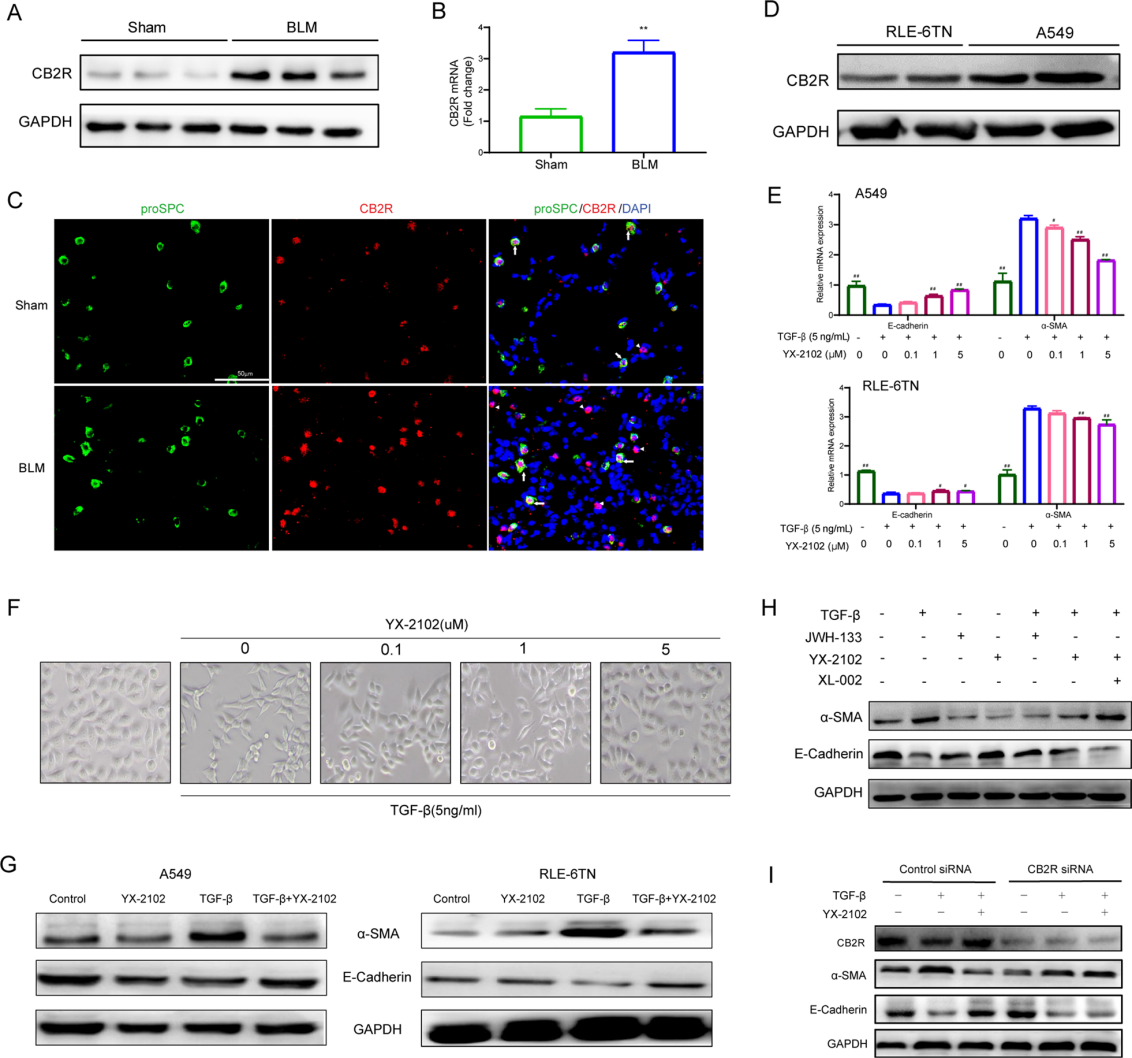
YX-2102 retarded TGF-β1-induced EMT in a CB2 R-dependent pathway. Rats were intratracheally treated with 5 mg/kg BLM once at day 0, followed by YX-2102 or vehicle treatment by intraperitoneal injection at a dose of 25 mg/kg daily. On day 21, rats were sacrificed and sampled. **A**, **B** Western blotting and qPCR were performed to evaluate the expression of CB2R in rats’ lung tissues of bleomycin-induced pulmonary fibrosis and sham group. **C** Immunofluorescence staining for Pro-SPC (green) and CB2R (red) in lung tissues. Arrows indicate epithelial cells with CB2R positive aggregates. Arrowheads indicate inflammatory cells with CB2R positive aggregates. Scale bar = 50 μm. **D** The expression of CB2R in A549 and RLE-6TN cells was evaluated by WB. **E** Cells were pretreated with 0.1, 1.0 or 5.0 µM indicated compounds for 2 h, followed by stimulation with 5 ng/mL TGF-β1 for another 48 h. DMSO treatment was set as control. The mRNA levels of E-cadherin and α-SMA were analyzed by qRT–PCR. **F** The morphological changes of A549 cells were observed at 48 h (magnification ×200). **G** Western blotting was performed to analyze the expression of E-cadherin and α-SMA in A549 (left panel) and RLE-6TN cells (right panel). **H** A549 cells were treated with YX-2102, JWH-133 or XL-002 for 2 h and stimulated with 5 ng/mL TGF-β1 for another 48 h. The levels of a-SMA and E-cadherin was assessed by western blotting. **I** Cells were transfected with 10 nM of control (scrambled) siRNA, CB2R-siRNA, subsequently pretreated with YX-2102 (5.0 µM) for 2 h, followed by TGF-β1 (5 ng/mL) treatment for 48 h. The expression of CB2R, α-SMA and E-cadherin was assessed by western blotting. Data are expressed as mean ± SEM, n = 5, ^**^P < 0.01 versus the Sham group, ^#^P < 0.05, ^##^P < 0.01 versus the TGF-β group (TGF-β = 5 ng/mL, YX-2102 = 0 µM)

This study first examined CB2R expression during lung fibrosis to investigate the correlation between YX-2102 treatment and CB2R function. The western blot (Fig. 4A) and qRT-PCR (Fig. 4B) showed that the CB2R expression was upregulated at mRNA and protein levels in BLM-induced rat PF lung tissue, compared with the sham control group. This study then assessed the cellular source of CB2R protein expression in the lungs. Immunofluorescence was used to examine the expression of surfactant-associated protein C (pro-SPC), a specific marker for AECIIs (type II alveolar epithelial cells), and CB2R in lung tissue to verify CB2R expression in alveolar epithelial cells in vivo. A clear colocalization of CB2R with pro-SPC was observed in alveolar epithelial cells (Fig. 4C). Meanwhile, infiltrating inflammatory cells in pulmonary interstitium and alveolar spaces also indicated strong CB2R positive signals. These features are consistent with the latest literature indicating that CB2R in the lung tissues could be a signature of PF [41]. Furthermore, the expression of CB2R was examined in A549 (human type II alveolar epithelial cell line) and RLE-6TN (rat alveolar type II cell line). CB2R was significantly upregulated in both cell lines (Fig. 4D).

The above results suggested that YX-2102 can alleviate lung fibrosis by regulating epithelial-mesenchymal transition (EMT). Therefore, this study investigated whether YX-2102 can inhibit the EMT process. Briefly, A549 and RLE-6TN cells were pretreated with 0.1, 1.0 or 5.0 µM YX-2102 for 2 h, followed by stimulation with 5 ng/mL TGF-β1 for 48 h to evaluate the effect of YX-2102 on TGF-β-induced EMT in alveolar epithelial cells. The mRNA expression of mesenchymal phenotype marker α-SMA increased, while the epithelial phenotype marker E-cadherin decreased (Fig. 4E). However, increasing doses of YX-2102 significantly increased the mRNA levels of E-cadherin and significantly suppressedα-SMA expression. YX-2102 prevented EMT induced by TGF-β1 in a dose-dependent manner. Consistently, light microscopy showed that YX-2102 pretreatment (5.0 µM) blocked the TGF-β1-induced spindle-shaped morphological change and A549 cells maintained their epithelial pebble-like morphology (Fig. 4F). Then, E-cadherin and α-SMA expression at protein levels were measured to further verify the effect of YX-2102 on EMT development. YX-2102 pretreatment increased the expression of E-cadherin and decreased α-SMA and fibronectin expression in A549 and RLE-6TN cells treated by TGF-β1 (Fig. 4G). Collectively, these results indicate that YX-2102 is a new potential EMT inhibitor.

To study the link between YX-2102 and CB2R in TGF-β1-induced EMT, the effect of YX-2102 was examined in A549 cells in presence of CB2R agonist (JWH-133) or antagonist (XL-002) [15]. As expected, both YX-2102 and JWH-133 treatment increased the expression of E-cadherin and decreased α-SMA expression in TGF-β1–treated A549 cells, while the effect of YX-2102 was reverted in the presence of CB2 antagonist (XL-002, 5.0 µM) (Fig. 4H). To intensively investigate whether inhibitory effect of YX-2102 on TGF-β1-induced EMT was directly related to CB2R activation, we use specific siRNA targeting CB2R to downregulate the expression of CB2R in A549 cells. As shown in Fig. 4I, YX-2102 could reduce the levels of a-SMA and increase the expression of E-cadherin in A549 cells treated by TGF-β1. While, this effect was nearly abolished by CB2R knockdown (Fig. 4I). These data imply that YX-2102 inhibits TGF-β1-induced EMT via a CB2R-dependent pathway.

### YX-2102 suppressed TGFβ1-Smad2/3 signaling pathway in A549 cells

**Fig. 5:**
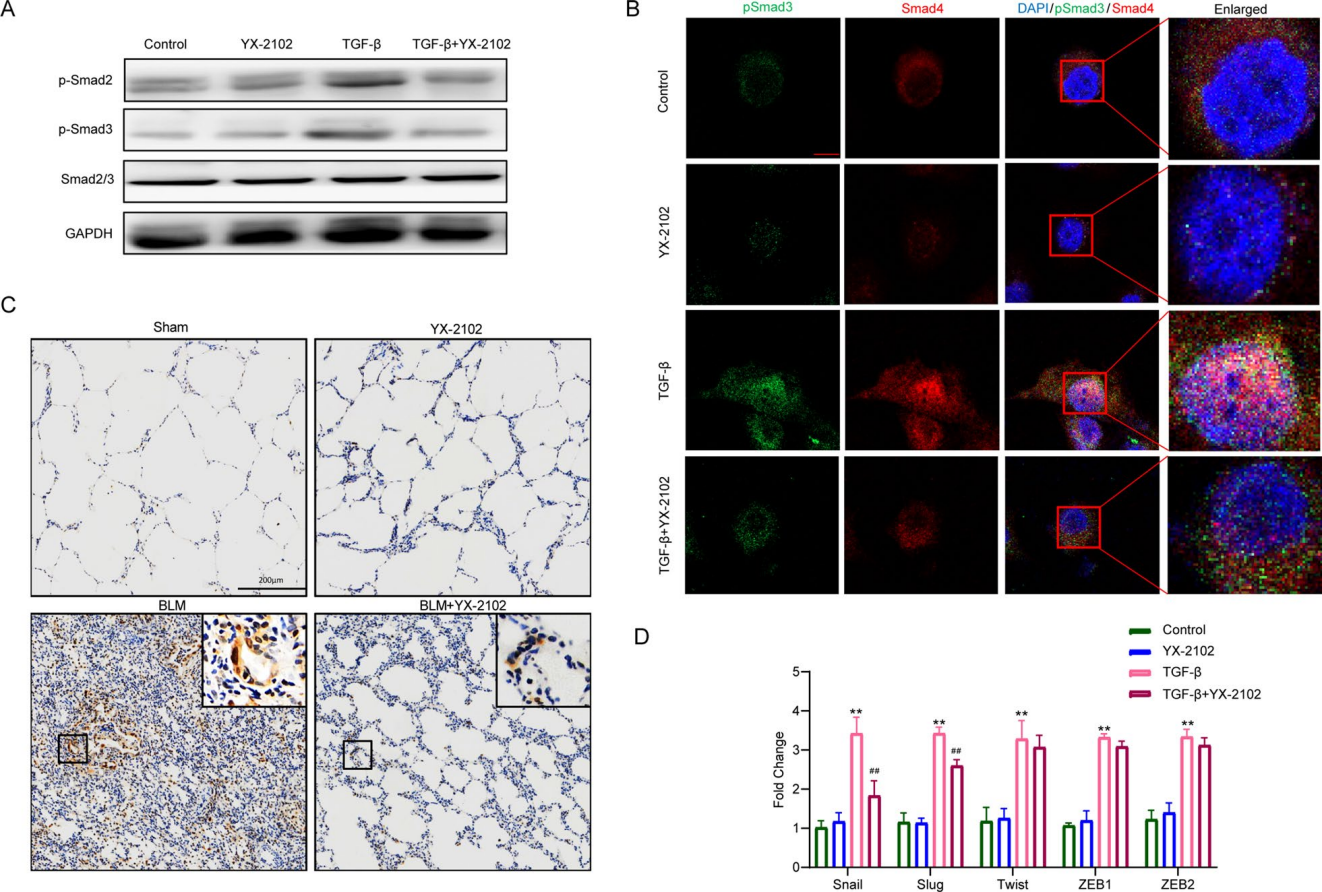
YX-2102 suppressed TGFβ1-Smad2/3 signaling and decreased EMT-associated transcription factors. A549 cells were pretreated with YX-2102 (5.0 µM) for 2 h and stimulated with TGF-β1 (5 ng/mL) for 24 h. Control cells were treated with DMSO. **A** WB analyses of p-Smad2/3 expression. **B** Confocal analysis of A549 cells stained with pSmad3 (green) and Smad4 (red) antibodies after TGF-β1 stimulated with/out YX-2102. Nuclei were counter-stained with DAPI (blue). Scale bar = 10 μm. **C** IHC evaluating the expression of p-Smad3 in the lungs of rats at 21 days (magnification ×200). Scale bar = 200 μm. **D** A549 cells (untreated or treated with 5.0 µM YX-2102 for 24 h), were stimulated or unstimulated with TGF-β1 (5 ng/mL). mRNA levels of slug, snail, ZEB-1, and twist were then determined using RT-qPCR. Data are expressed as mean ± SEM of three independent experiments. **P < 0.01 versus Sham groups. ^##^P < 0.01 versus the TGF-β group

TGF-β signaling pathway plays a key role in the pathogenesis of PF. To elucidate the molecular mechanisms through which YX-2102 suppresses TGF-β-induced-EMT and lung fibrosis, we used western blotting and RT-qPCR analyses to evaluate changes in downstream mediators of TGF-β signaling as well as several EMT transcription factors. As anticipated, phosphorylated Smad2 and Smad3 (p-Smad2/3) significantly increased upon stimulation with TGF-β1, and pretreatment with YX-2102 markedly retarded the phosphorylation of Smad2 and Smad3 (Fig. 5A). These phosphorylated Smads then form a complex with Smad4 to activate transcriptional responses in the nucleus. Thus, to determine the subcellular localization of pSmad3/Smad4 proteins, the pSmad3–Smad4 complex was detected using specific antibodies against pSmad3 and Smad4. The results showed that YX-2102 pretreatment obviously inhibited TGF-β-induced nuclear translocation of pSmad3 and Smad4 (Fig. 5B). Moreover, immunohistochemical staining revealed that the BLM group had higher levels of phosphorylated Smad3 when compared with the sham group, and that YX-2102 inhibits Smad3 phosphorylation (Fig. 5C).

Various crucial transcription factors such as Snail, Slug, Twist, ZEB1 and ZEB2 have been shown to participate in EMT progression. To gain more insights into the mechanisms via which YX-2102 prevents TGF-β1-induced EMT, RT-qPCR was used to assess its effects on the expression of the transcription factors mentioned above and found them to be significantly elevated 24 h after TGF-β1 stimulation. Notably, treatment with YX-2102 suppressed the levels of Snail and Slug mRNA while modestly affecting the expression of Twist, ZEB1, and ZEB2 mRNA (Fig. 5D), which is consistent with past findings [39]. Ravi et al. found that JWH-015, a CB2R agonist, suppressed the expression of Snail and Slug in TGF-β1-induced EMT in A549 cells. Collectively, these data suggest that YX-2102 inhibits YX-TGF-β1-induced EMT in A549 cells by inhibiting TGFβ1-Smad2/3 signaling and associated transcription factors.

### YX-2102 promoted the activation of Nrf2-Smad7 pathway

**Fig. 6:**
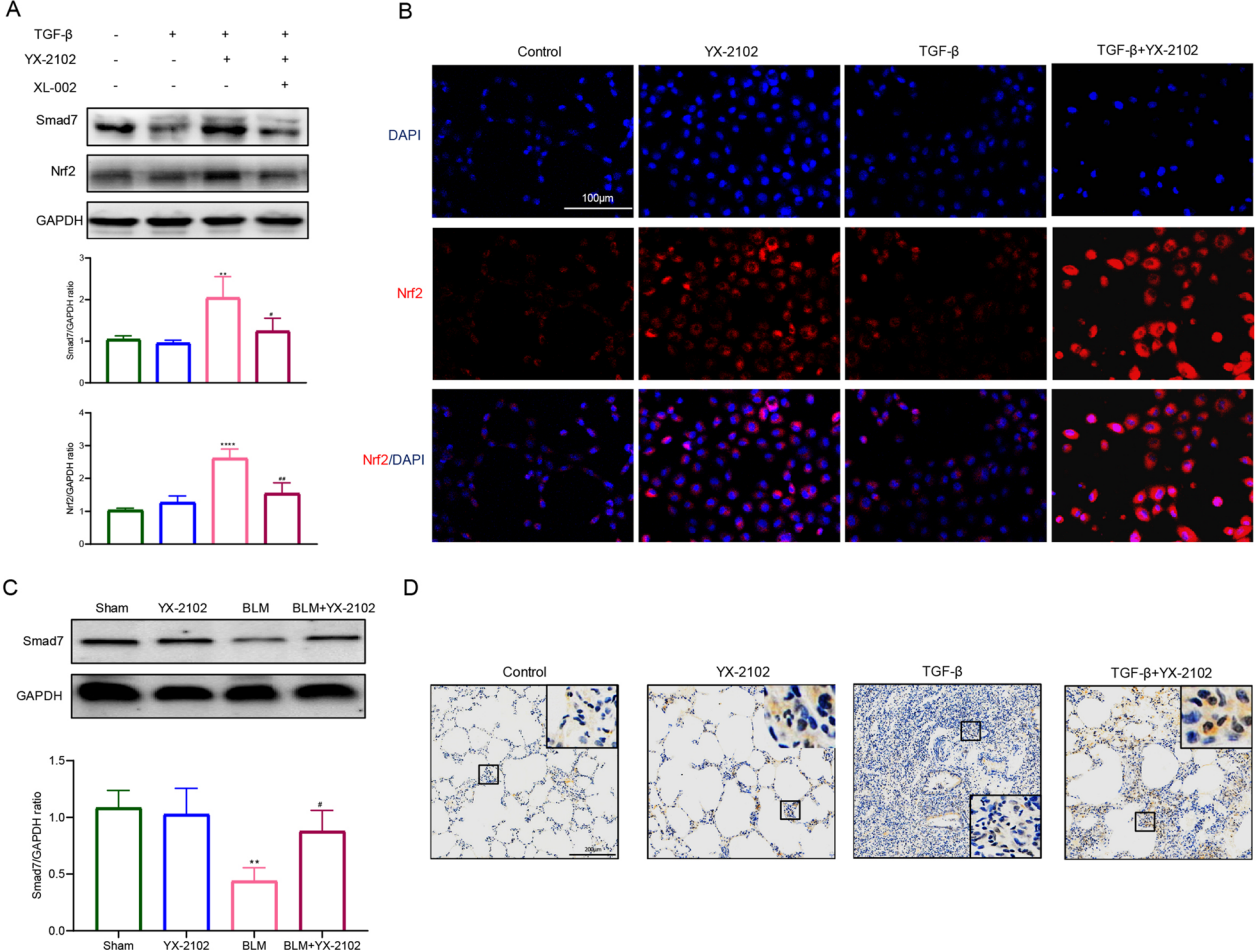
YX-2102 enhanced Nrf2-Smad7 pathway activation in A549 cells. A549 cells pretreated with YX-2102 (5.0 µM), with or without 5 µM XL-002 (a CB2R antagonist) for 2 h, were subjected to TGF-β1 stimulation (5 ng/mL) for another 24 h. **A** Western blot analyses of Smad7 and Nrf2 expression. **B** Immunofluorescence analysis of Nrf2 (red). Nuclei were counter-stained with DAPI (blue). Scale bar = 100 μm. **C** Western blot analyses of Smad7 expression. **D** Immunohistochemical analysis of Smad7 expression in the lungs of 21-day-old rats (magnification ×200). Large boxes are magnified views of the small boxes. Scale bar = 200 μm. Data are expressed as mean ± SEM of three independent experiments

Smad7 is an inhibitor of TGF-β1/Smad signaling. To assess if YX-2102 interrupts TGF-β1 signaling in lung epithelial cells by regulating Smad7 expression, we examined the levels of Smad7 in A549. Notably, reduced Smad7 levels were observed in A549 cells upon TGF-β1 stimulation, and this effect was prevented by pretreatment with YX-2102 (Fig. 6A). Furthermore, YX-2102 treatment markedly attenuated oxidative stress in rats with BLM-induced pulmonary fibrosis, indicated by reduced MDA levels, enhanced SOD activity, and increased GSH content (Additional file 1: Fig. S5). Nuclear factor erythroid 2-related factor 2 (Nrf2) is a key factor in oxidative stress and past studies show that CB2R activation may functionally enhance Nrf2 expression, which may modulate Smad7 expression [8, 42]. WB analysis demonstrated that YX-2102 significantly upregulated the expression of Nrf2 in A549 cells stimulated with TGF-β1 (Fig. 6A). IF analysis revealed that Nrf2 primarily localized in the cytoplasm of control A549 cells. However, treatment with YX-2102 triggered Nrf2 translocation into the nucleus (Fig. 6B). The effects of YX-2102 on Nrf2-Smad7 signaling in A549 cells were inhibited by pretreatment with XL-002 (5.0 µM), a CB2R antagonist (Fig. 6A). IHC and WB analysis revealed that relative to the sham group, Smad7 expression was significantly lower in the BLM group, and YX-2102 enhanced Smad7 expression (Fig. 6C, D). These data indicate that YX-2102 might inhibit EMT in A549 cells partly through CB2 receptor-mediated Nrf2-Smad7 elevation.

## Discussion

Previous studies have identified numerous CB2R ligands with different scaffolds, including the natural polyphenol compound, tetrahydromagnolol [11], polycyclic chromene JWH-133 [5], indole derivative AM630 [12], and substituted imidazole, SR144528 [15]. These agonists or antagonists have demonstrated good binding affinity with CB2R and pharmacological activities against various pathologies, including fibrosis and non-small cell lung cancer [39]. These studies demonstrated the therapeutic potential of developing new CB2R ligands for the treatment of several diseases.

Here, we identified a novel CB2R agonist YX-2102 through in silico screening of an in-house library consisted of over 1600 + molecules with high structural diversity and complexity. The structure of YX-2102, which contains a pyrano[2,3-*b*]pyridine scaffold with continuous stereocenters, is rarely found in commercial compound libraries. Similarity comparison between YX-2102 and 1000 high affinitive CB2R ligands was less than 50%, suggesting it is quite different from the reported ligands in scaffolds (Additional file 1: Fig. S6). Despite its high molecular complexity and unusual structural features, YX-2102 can be easily synthesized via well-established routes, with high yield and good enantioselectivity. Hence, where structural optimization is needed, YX-2102 is easily modifiable to obtain derivatives with better activity. These results also demonstrated the superiority and potential of the organic synthetic methodology-based library in drug lead discovery.

We also showed that YX-2102 administration significantly ameliorated BLM-induced PF in rats, and alleviated early inflammatory response induced by bleomycin by inhibiting M1 macrophage polarization. Moreover, our results indicate that YX-2102 retarded TGF-β1-induced EMT in alveolar epithelial cells in a CB2R-dependent manner, at least partially by enhancing Nrf2-mediated Smad7 elevation, highlighting CB2R as a potential therapeutic target against fibrotic diseases.

Mounting evidence has implicated the cannabinoid system in lung homeostasis and disease [43]. CB1R and CB2R are thought to have different and sometimes opposing roles in the development of tissue fibrosis [2, 44]. The CB1R is mainly present in the central nervous system while the CB2R with a dynamic range of expression levels in different cell types of human tissues, including immune and hematopoietic cells, epithelial cells, myocytes, fibroblasts, and skin keratinocytes [45]. Hence CB2R is considered a promising therapeutic target in the treatment of various diseases associated with inflammation and tissue injury. However, few studies have elucidated the role of cannabinoid receptors in PF. Pharmacologic inhibition of CB1R enhances radiation- [46] and BLM-induced pulmonary fibrosis [44]. In contrast, recent studies show that CB2R activation using a specific agonist, JWH133, suppressed nicotine-induced mouse interstitial lung fibrosis [47] and BLM-induced pulmonary fibrosis [6]. Consistently, using YX-2102, a novel selective CB2R agonist, we show that CB2R activation protects against BLM-induced lung fibrosis. Past studies identified cannabinoid receptors on structural cells and most inflammatory cells in the lung [43]. Our data show that CB2R is highly expressed in alveolar epithelial cells and in bleomycin-induced PF rat lung tissue, especially in the fibrotic area. Taken together, these data implicate CB2R in PF progression and highlight it as a promising target for the identification of novel therapies against PF.

Numerous studies show that CB2R activation exhibits anti-inflammatory effects and it has been recognized as a potential target for several inflammatory diseases [7, 48]. Sustained inflammation plays a key role in the pathogenesis of pulmonary fibrosis. Inflammatory cells in the lungs, including lymphocytes, neutrophils, and macrophages, are important sources of various inflammatory mediators and influence the onset and progression of PF. Intratracheal instillation of bleomycin causes acute lung injury, and the ensuing inflammatory response is implicated in fibrosis. Here, we found that intrapulmonary exposure to bleomycin markedly enhances the number of inflammatory cells and associated inflammatory mediators, while YX-2102 administration alleviates the inflammatory responses induced by bleomycin. Notably, the expression of the anti-inflammatory cytokines was significantly enhanced by YX-2102. These results reaffirmed the anti-inflammation properties of CB2R during the early stages of pulmonary fibrosis.

Nevertheless, the mechanism by which CB2R modulates inflammatory responses during pulmonary fibrosis had not been determined. In the airway and lung microenvironment, macrophages are intricately involved in inflammation and fibrosis. Infiltrating macrophages at sites of lung tissue injury were activated and polarized into M1 or M2 subpopulations, with M1 macrophages being pro-inflammatory/anti-fibrotic and M2 macrophages being pro-fibrotic or regulatory [40]. It has been reported that the anti-inflammatory effect of CB2R may be mediated by regulating macrophage polarization. Numerous studies indicate that CB2R activation attenuates inflammation via reducing M1 macrophage polarization and enhancing M2 polarization. In contrast, Du et al. reported that CB2R alleviates inflammation by suppressing M1 macrophages, rather than upregulating M2 macrophages [36]. Our data have shown that YX-2102 markedly decreased the number of M1 macrophages and increased M2 macrophages. However, because M2 macrophages promote tissue fibrosis and CB2R inhibits fibrosis, M2 polarization upon CB2R activation seems counterintuitive. There are several potential explanations for this. (i) Excessive inflammatory responses in the early stages of BLM-induced lung injury/fibrosis that aggravate tissue damage. Thus, reduction of early infiltrating pro-inflammatory M1 macrophages upon CB2R activation may mitigate the severity of subsequent fibrosis. (ii) The current classification of M1/M2-polarized macrophages may be overly simplistic. Actually, both M1 and M2 macrophages are intricately involved in the progression of PF and their contributions to this disease remain elusive. (iii) A timely switch and dynamic balance of M1 and M2 macrophages are needed to maintain tissue homeostasis. It may be presumed that CB2R activation by YX-2102 suppresses the accumulation of M1 macrophages, which in turn, led to enhancement of the relative proportion of M2 macrophages in the lung. These speculations will be the focus of future investigations. Our findings indicate that YX-2102 may improve lung fibrosis via CB2R-mediated inhibition of M1 polarization, thereby significantly reducing early lung inflammation.

EMT plays a critical role in pathogenesis of fibrosis in many organs, including lung [49, 50]. It is a highly active process where epithelial cells lose their epithelial E-cadherin and gains mesenchymal markers such as fibronectin and α-SMA [51]. Fibronectin is required for collagen matrix assembly and α-SMA is an important biomarker of activated myofibroblasts, both of which were believed to be the key contributors to organ fibrosis [52]. Following YX-2102 treatment, the decreased expression of fibronectin and α-SMA concomitant with an elevation in E-cadherin levels implied that YX-2102 might ameliorates the degree of lung fibrosis through inhibiting the process of EMT. Furthermore, of multiple stimuli involved in pulmonary fibrosis, TGF-β1 is considered the master regulator of pathological fibrosis and is a widely studied profibrotic factor involved in driving EMT [53]. The TGF-β1/Smad pathway has been implicated in the progression of PF. Upon binding with its receptor, TGF-β1 triggers the phosphorylation of Smad2/3 for their activation. The Smad2/3 dimer and Smad4 form complexes and then translocate into the nucleus. In nucleus, the Smad complex suppresses the expression of E-cadherin through transcription factors Snail1 and Slug. The transcriptional effects of TGF-β/Smad signaling also indirectly drive EMT by inducing the expression of Twist, Zeb1 and Zeb2. These events result in downregulation of epithelial markers and the upregulation of mesenchymal genes [54]. However, the involvement of CB2R and its role in TGF-β1-induced EMT have not been previously studied. Here, we show that CB2R activation by YX-2102 significantly repressed changes in cellular EMT markers by reducing the activation Smad2/3 and inhibiting the translocation of Smad3:Smad4 complex into the nucleus in response to TGF-β1 stimulation. Additionally, YX-2102 markedly reduced the mRNA levels of the EMT transcription factors, Snail and Slug. These results are consistent with observations that in BLM-induced PF rats, EMT-related markers and TGF-β1/Smad signaling was suppressed by YX-2102. Consistently, JWH-015, another CB2R agonist, is reported to inhibit macrophage-induced EMT in A549 cells by downregulating epidermal growth factor receptor (EGFR) and its targets [39]. Thus, we presumed that YX-2102, a CB2R agonist may inhibit EMT by inducing biological mediators that disturb TGF-β/Smad signaling, which may account for the fibrosis alleviation.

To investigate the mechanism by which YX-2102 inhibits TGF-β1-induced EMT, we assessed the level of Smad7, a negative regulator of TGF-β-signaling. Smad7 inhibits TGF-β-induced transcriptional responses by inhibiting TGF-β-mediated Smad2/3 phosphorylation or interfering with Smad-DNA interaction. Decreased Smad7 expression in fibrotic lung tissues corresponds with increased TGF-β1/Smad pro-fibrotic signaling, which is a critical event in PF [55]. Our results are consistent with past findings that Smad7 is significantly suppressed in a bleomycin-induced PF mouse model. We also found that YX-2102 restored Smad7 expression in TGF-β treated alveolar epithelial cell in a CB2-dependent manner, in vitro. Correspondingly, treatment with YX-2102 significantly elevated the level of Smad7 in lung tissues of rats after bleomycin instillation. A past study found that CB2R activation markedly increased the level of Smad7 during skin wound repair in mice [56]. To our knowledge, this is the first study linking CB2 activation to induction of Smad7 in alveolar epithelial cells.

Nrf2 is a redox-sensitive transcription factor that protects against oxidative stress injury and inflammation. Studies have shown that Nrf2 protects from PF. Nrf2 has been reported to protect against PF by regulating cellular redox levels [57]. Additionally, Nrf2-mediated redox imbalance promotes profibrotic myofibroblast phenotypes, resulting in persistent fibrosis in lungs of aged mice [58]. Given the effect of CB2R on oxidative stress, we investigated if YX-2102 influences Nrf2 expression in alveolar epithelial cells, and found that activating CB2R with YX-2102 markedly enhanced Nrf2 expression and promotes Nrf2 translocation into the nucleus. These effects were inhibited using a selective CB2R antagonist. Numerous studies have reported crosstalk between CB2R and Nrf2 signaling pathway [8, 42]. CB2R activation ameliorates myocardial fibrosis by accelerating Nrf2 translocation into the nucleus and suppressing the TGF-beta1/Smad3 pathway in a Nrf2-dependent manner [42]. Interestingly, recent studies found that Nrf2 inhibits TGF-β1-induced EMT and lung fibrosis by regulating snail expression [59]. Furthermore, Nrf2 is reported to positively regulate Smad7 expression and the Nrf2-Smad7 axis plays a critical role in the prevention of renal and cardiac fibrosis [60, 61]. Thus, although the precise mechanisms remain to be explored, past studies and our findings imply that the inhibitory effect of CB2R activation with YX-2102 on TGF-β-induced EMT and pulmonary fibrosis might be partly ascribed to Nrf2-mediated smad7 elevation that serve to perturb the TGF-beta pathway during this process. However, this is a potential mechanism by which CB2R activation by YX-2102 inhibits EMT. Further in vitro and in vivo studies are needed to elucidate the mechanism underlying and to determine its feasibility in future applications.

## Conclusion

**Fig. 7:**
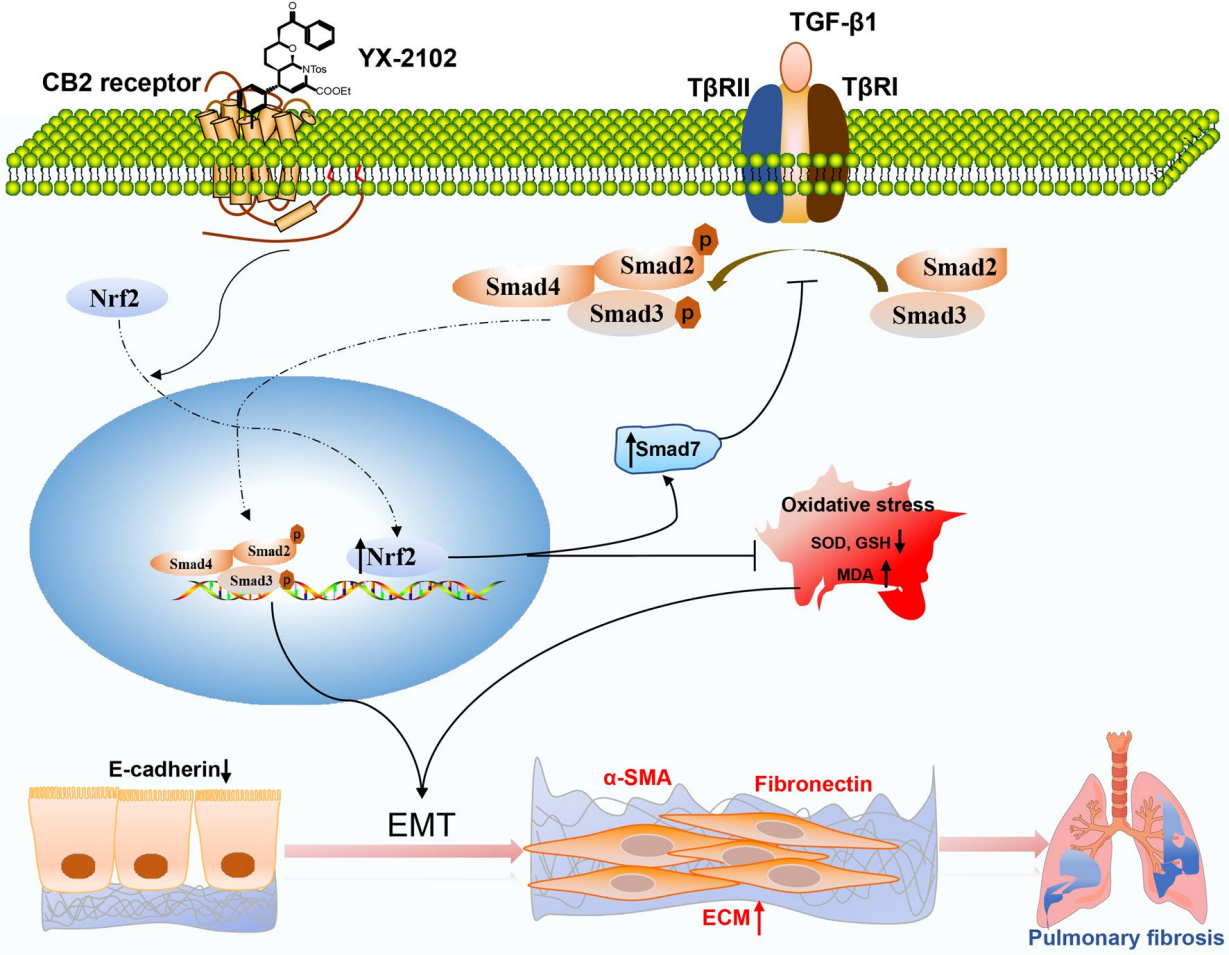
A schematic representation of the antifibrotic effect of YX-2102 in the inhibition of lung alveolar EMT. Activation of CB2R by YX-2102 treatment promotes the translocation of Nrf2 to the nucleus, which subsequently reduced oxidative stress and elevating Smad7 protein levels, thereby inhibiting Smad2/3 phosphorylation to block TGF-β signaling. These consecutive events result in EMT suppression in lung alveolar epithelial cells and ultimately attenuates bleomycin-induced pulmonary fibrosis

In conclusion, the present study has shown that YX-2102, a novel CB2R activator, alleviates bleomycin-induced pulmonary fibrosis in rats, probably by inhibiting inflammation during the early stage of PF, as well as by suppressing TGF-β1-induced EMT in a CB2R-dependent manner by enhancing Nrf2-mediated Smad7 elevation (Fig. 7). Our data indicate that CB2R and YX-2102 are potential target and candidate agent for the treatment of lung fibrosis, respectively.

## Supplementary Information

**Additional file 1:** **Figure S1.** Representative H&E-stained images of lung harvested from the rats after various treatments for 21 days. n = 5 or 6 per group. Scale bar=50 μm. **Figure S2.** Binding affinities of compounds YX-2102 (A), ZZ-4113 (B), XYC-4104 (C) and XYC-4106 (D). **Figure S3.** The representative gross-morphological images of rat lung from each group at day 21. **Figure S4.** The changes in body weight for each experimental group; Data are expressed as mean ± SEM. n = 5 rats per group. **P < 0.001 versus BLM group. **Figure S5.** YX-2102 improved the oxidative stress in rats with BLM-induced pulmonary fibrosis. Oxidative stress was assessed by measuring the activity of tissue superoxide dismutase (SOD) and the contents of malondialdehyde (MDA) and glutathione (GSH). The measurement was repeated three times and the data were represented as mean ± SD; ****P < 0.0001 versus sham group, #P < 0.05, ###P < 0.001 versus BLM group. **Figure S6.** Structure similarity map of CB2R ligand. 1000 CB2R ligand were collected from CHEMBL (https://www.ebi.ac.uk/chembl/) whose binding affinity was lower than 10 nM. The shorter distance means to the more obvious similarity. The structures are outliers if similarity to other structures is less than 50%, meaning the similarity of YX-2102 to other structure is less than 50%. **Table S1.** Purchase, dilution and storage conditions of primary and second antibodies. **Table S2.** Structural information and sources of the active molecules used in the manuscript. **Table S3.** Primers used for real-time qPCR (h and r indicate human and rat species, respectively). **Table S4.** Components of the binding free energy (kcal/mol) calculated by MM/GBSA approach

**Additional file 2:** **Video S1.** A Representative 3-dimensional (3D) structure and morphology of rat lung in the sham group at day 21 after drug administration.

**Additional file 3:** **Video S2.** A Representative 3D structure and morphology of rat lung in the YX-2102 group at day 21 after drug administration.

**Additional file 4:** **Video S3.** A Representative 3D structure and morphology of rat lung in the BLM group at day 21 after drug administration.

**Additional file 5:** **Video S4.** A Representative 3D structure and morphology of rat lung in the BLM+YX-2102 group at day 21 after drug administration.

## Data Availability

The datasets supporting the conclusions of this article are included within the article and supporting information.

## Abbreviations

α-SMA: alpha-smooth muscle actin
Arg-1: Arginase-1
BALF: Bronchoalveolar lavage fluid
BLM: Bleomycin
CB2R: Cannabinoid 2 receptor
DMSO: dimethyl sulfoxide
EGFR: epidermal growth factor receptor
EMT: Epithelial-to-mesenchymal transition
GAPDH: glyceraldehyde-3-phosphate dehydrogenase
Hyp: Hydroxyproline
iNOS: inducible nitric oxide synthase
Lym: lymphocytes
MCP-1: monocyte chemoattractant protein-1
MD: molecular dynamic simulation
MPO: myeloperoxidase
Mφ: macrophage
Neu: neutrophils
Nrf2: nuclear factor erythroid 2-related factor 2
PF: Pulmonary fibrosis
SDS-PAGE: sodium dodecyl sulfate-polyacrylamide gel electrophoresis
TGF-β1: transforming growth factor beta-1
RMSD: Root mean squared diviation
Δ9-THC: Δ9-tetrahydrocannabinol
ΔE_vdw_: ΔE_van der Waals_
