# Supplementary material for: Discovery of a pyrano[2,3-b]pyridine derivative YX-2102 as a cannabinoid receptor 2 agonist for alleviating lung fibrosis
Source: J Transl Med. 2022 Dec 6;20:565. doi: 10.1186/s12967-022-03773-1 (PMC9724349; doi:10.1186/s12967-022-03773-1)
Supplement: Supplementary file 1 — Additional file 1: Figure S1. Representative H&E-stained images of lung harvested from the rats after various treatments for 21 days. n = 5 or 6 per group. Scale bar=50 μm. Figure S2. Binding affinities of compounds YX-2102 (A), ZZ-4113 (B), XYC-4104 (C) and XYC-4106 (D). Figure S3. The representative gross-morphological images of rat lung from each group at day 21. Figure S4. The changes in body weight for each experimental group; Data are expressed as mean ± SEM. n = 5 rats per group. **P < 0.001 versus BLM group. Figure S5. YX-2102 improved the oxidative stress in rats with BLM-induced pulmonary fibrosis. Oxidative stress was assessed by measuring the activity of tissue superoxide dismutase (SOD) and the contents of malondialdehyde (MDA) and glutathione (GSH). The measurement was repeated three times and the data were represented as mean ± SD; ****P < 0.0001 versus sham group, #P < 0.05, ###P < 0.001 versus BLM group. Figure S6. Structure similarity map of CB2R ligand. 1000 CB2R ligand were collected from CHEMBL (https://www.ebi.ac.uk/chembl/) whose binding affinity was lower than 10 nM. The shorter distance means to the more obvious similarity. The structures are outliers if similarity to other structures is less than 50%, meaning the similarity of YX-2102 to other structure is less than 50%. Table S1. Purchase, dilution and storage conditions of primary and second antibodies. Table S2. Structural information and sources of the active molecules used in the manuscript. Table S3. Primers used for real-time qPCR (h and r indicate human and rat species, respectively). Table S4. Components of the binding free energy (kcal/mol) calculated by MM/GBSA approach [file 12967_2022_3773_MOESM1_ESM.docx]

Additional Information

**Discovery of a pyrano[2,3-*b*]pyridine derivative YX-2102 as** **a cannabinoid receptor 2 agonist for alleviating** **lung fibrosis**

**This file includes:**

Additional Video Legends

Additional Figures 1-5

Additional Tables 1-4

Additional Video Legends

Video S1.

A Representative 3-dimensional (3D) structure and morphology of rat lung in the sham group at day 21 after drug administration.

Video S2.

A Representative 3D structure and morphology of rat lung in the YX-2102 group at day 21 after drug administration.

Video S3.

A Representative 3D structure and morphology of rat lung in the BLM group at day 21 after drug administration.

Video S4.

A Representative 3D structure and morphology of rat lung in the BLM+YX-2102 group at day 21 after drug administration.


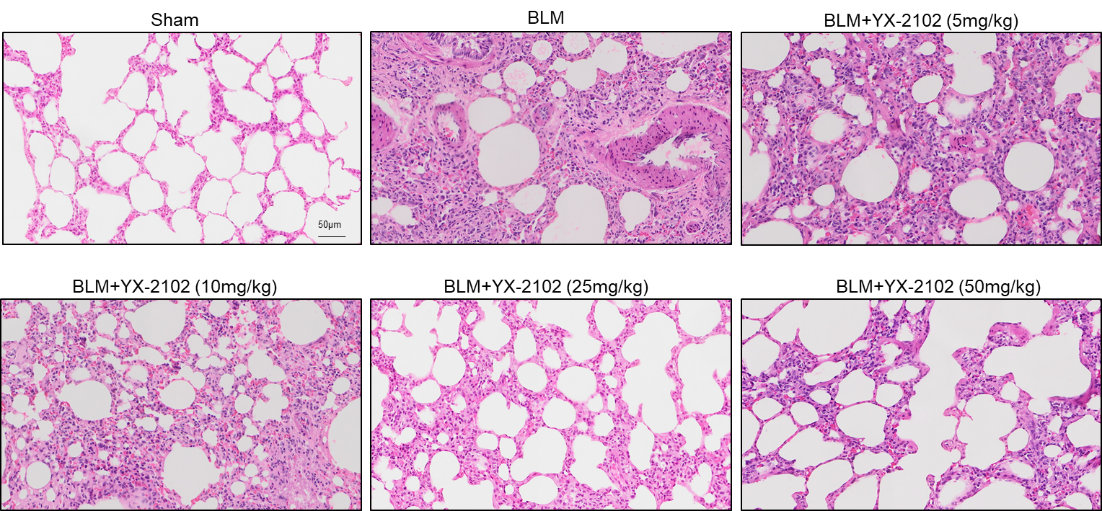


Figure S1. Representative H&E-stained images of lung harvested from the rats after various treatments for 21 days. n = 5 or 6 per group. Scale bar=50 μm.

| **A** | **B** |
| --- | --- |
| 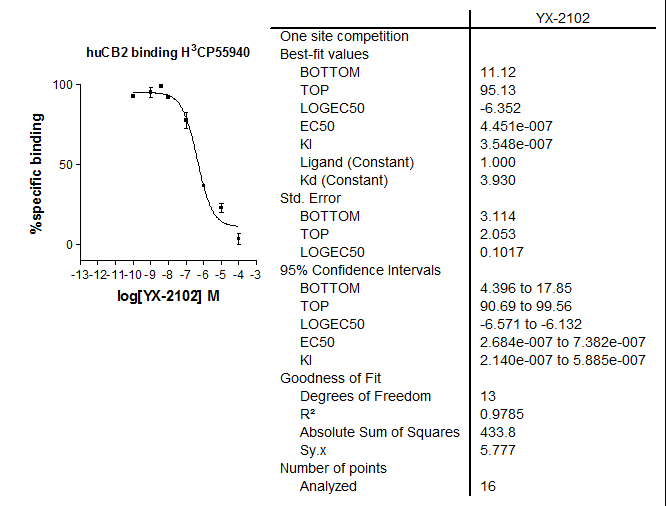 | 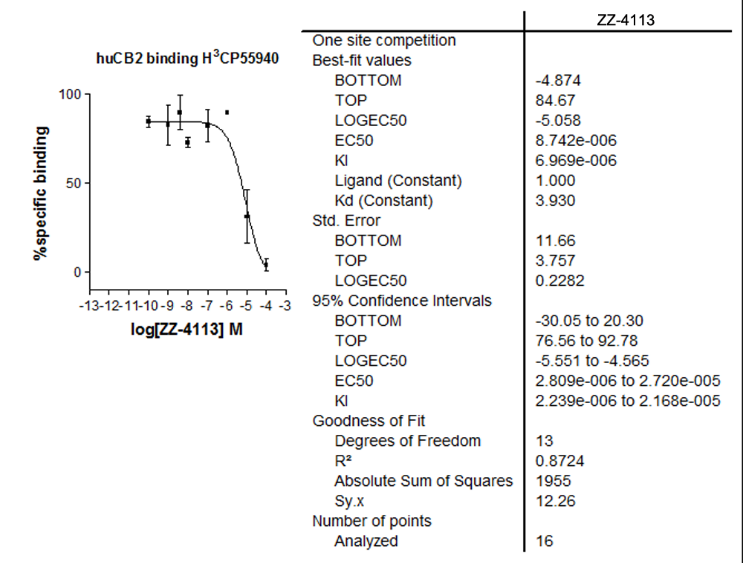 |
| **C** | **D** |
| 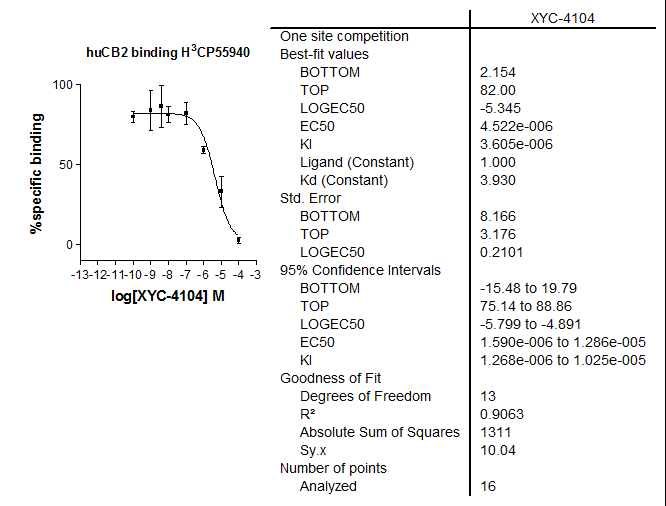 | 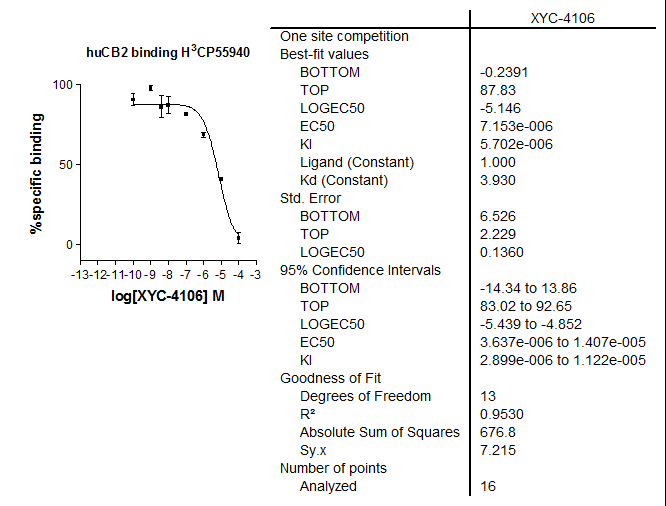 |

Figure S2. Binding affinities of compounds YX-2102 (A), ZZ-4113 (B), XYC-4104 (C) and XYC-4106 (D)


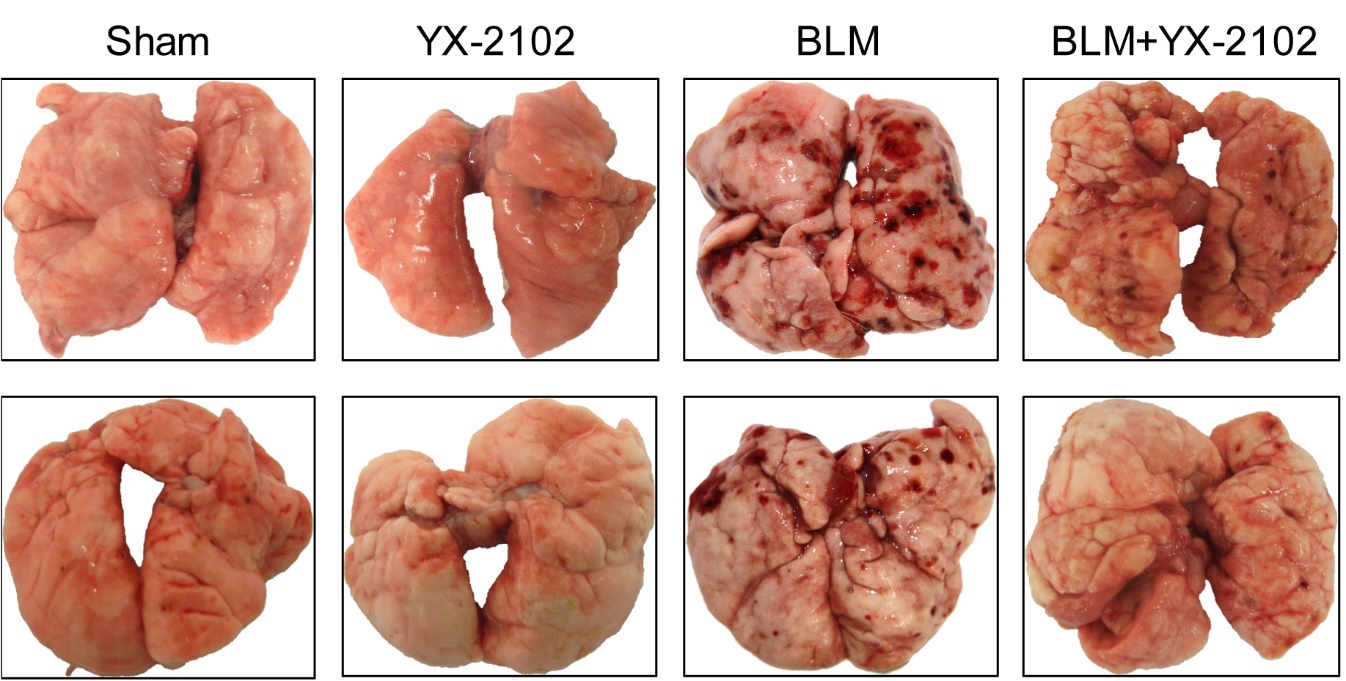


Figure S3. The representative gross-morphological images of rat lung from each group at day 21.


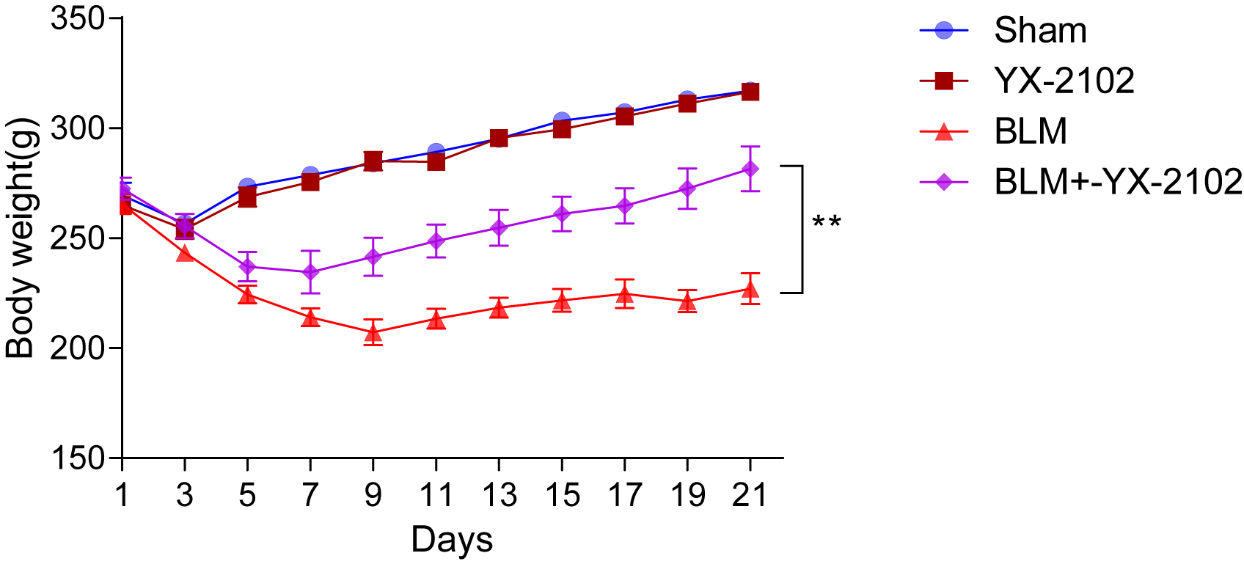


Figure S4. The changes in body weight for each experimental group; Data are expressed as mean ± SEM. n = 5 rats per group. **P < 0.001 versus BLM group.


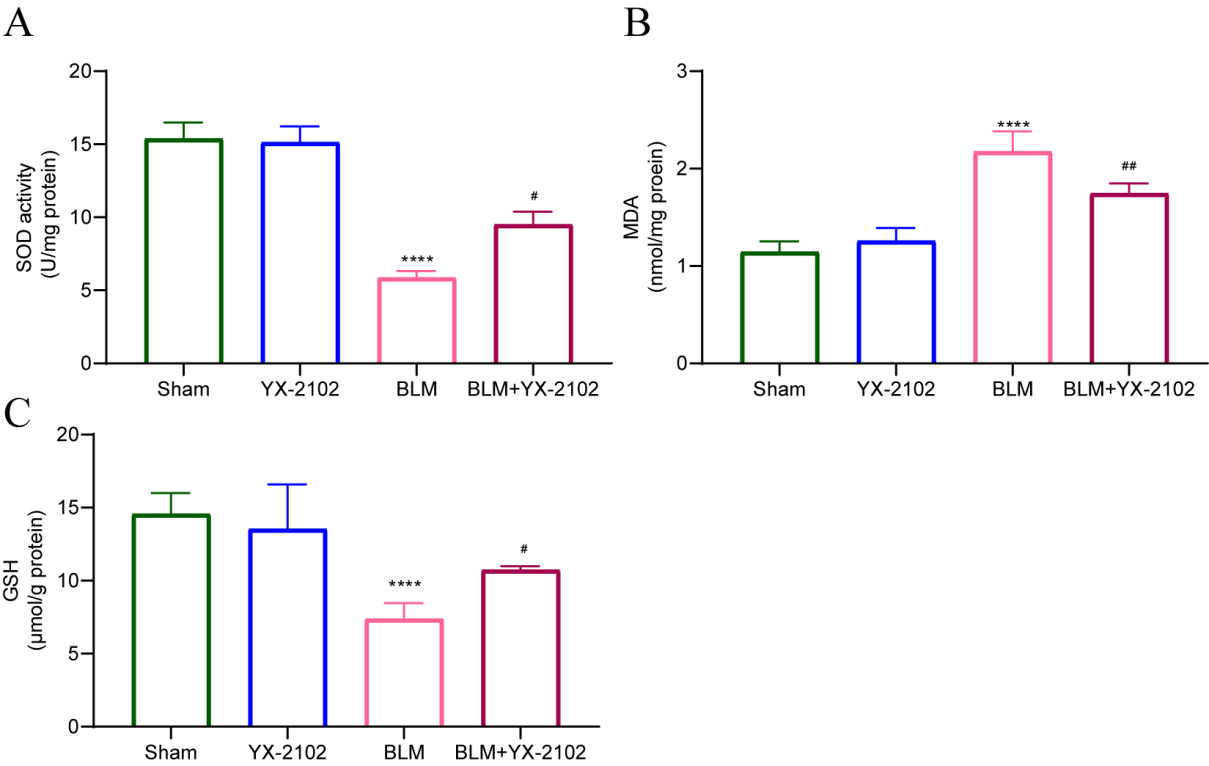


Figure S5. YX-2102 improved the oxidative stress in rats with BLM-induced pulmonary fibrosis. Oxidative stress was assessed by measuring the activity of tissue superoxide dismutase (SOD) and the contents of malondialdehyde (MDA) and glutathione (GSH). The measurement was repeated three times and the data were represented as mean ± SD; ****P < 0.0001 versus sham group, #P < 0.05, ###P < 0.001 versus BLM group.


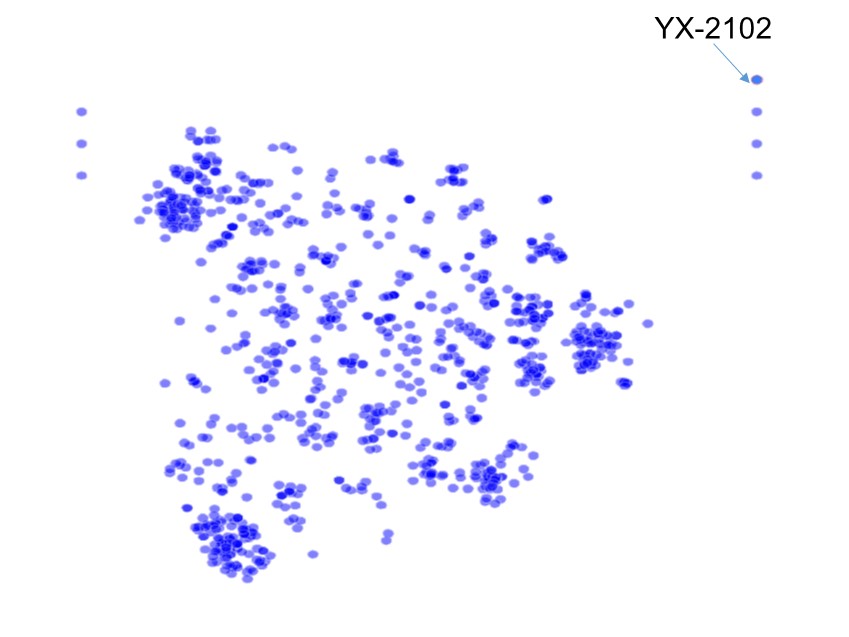


Figure S6. Structure similarity map of CB2R ligand. 1000 CB2R ligand were collected from CHEMBL (https://www.ebi.ac.uk/chembl/) whose binding affinity was lower than 10 nM. The shorter distance means to the more obvious similarity. The structures are outliers if similarity to other structures is less than 50%, meaning the similarity of YX-2102 to other structure is less than 50%.

Table S1. Purchase, dilution and storage conditions of primary and second antibodies.

| **Antibody** | **Cat.NO** | **Corporation** | **Dilution** | | **Storage** |
| --- | --- | --- | --- | --- | --- |
|  |  |  | **WB** | **IF/IHC** |  |
| TGF-β1 | ab92486 | Abcam | 1:500 | 1:400 | -20 ºC |
| α-SMA | ab5694 | Abcam | 1:300 | 1:400 | -20 ºC |
| E-cadherin | sc-8426 | Santa Cruz | 1:200 | 1:100 | 4 ºC |
| Fibronectin | sc-8422 | Santa Cruz | 1:200 | 1:100 | 4 ºC |
| CB2R | ab3561 | Abcam | 1:500 | 1:200 | -20 ºC |
| CD206 | GB13438 | Servicebio | - | 1:200 | -20 ºC |
| CD68 | ab31630 | Abcam | - | 1:200 | -20 ºC |
| MPO | ab65871 | Abcam | - | 1:200 | -20 ºC |
| iNOS | PA1-036 | Invitrogen | - | 1:200 | -20 ºC |
| pSmad2 | ab188334 | Abcam | 1:1000 | - | -20 ºC |
| pSmad3  pSmad3  Smad4 | 9520  bsm-33357M  46535 | CST  Bioass  CST |  | 1:200  1:500 | -20 ºC  -20 ºC  -20 ºC |
| Smad2/3 | sc-398844 | Santa Cruz |  |  | 4 ºC |
| Smad7 | 42-0400 | Invitrogen | 1:1000 | 1:500 | -20 ºC |
| Nrf2 | ab89443 | Abcam | 1:1000 | 1:500 | -20 ºC |
| GAPDH | sc25778 | Santa Cruz | 1:500 |  | 4 ºC |
| HRP-linked Antibody | sc2357 | Santa Cruz | 1:1000 | - | 4 ºC |

Table S2. Structural information and sources of the active molecules used in the manuscript.

| **Compound** | **Structure** | **Source** |
| --- | --- | --- |
| JWH-133 |  | Purchased from Sigma-Aldrich |
| XL-002 |  | Synthesized according to *ACS Med. Chem. Lett.* **2013**, *4*, 387. |
| ZZ-4113 |  | Synthesized according to *Org. Lett.* **2014**, *16*, 2370 |
| XYC-4102 |  | Synthesized according to *Org. Lett.* **2014**, *16*, 3208 |
| XYC-4104 |  |  |
| XYC-4106 |  |  |
| YX-2102 |  | Synthesized according to *Chin. J. Chem.* **2012**, *30*, 2669 |

Table S3. Primers used for real-time qPCR (h and r indicate human and rat species, respectively).

| Gene | Forward (5' to 3') | Reverse (5' to 3') |
| --- | --- | --- |
| Fibronectin (r) | TCGCCATCAGTAGAAGGTAGCA | TGTTATACTGAACACCAGGTTGCA |
| E-cadherin (h) | TGCACCAACCCTCATGAGTG | GTCAGTATCAGCCGCTTTCAG |
| E-cadherin (r) | GGGTTGTCTCAGCCAATGTT | CACCAACACACCCAGCATAG |
| α-SMA (h) | GACCGAATGCAGAAGGAGAT | CCACCGATCCAGACAGAGTA |
| α-SMA (r) | AGCCAGTCGCCATCAGGAAC | GGGAGCATCATCACCAGCAAAG |
| Slug (h) | GAGCATTTGCAGACAGGTCA | ACAGCAGCCAGATTCCTCAT |
| Snail1 (h) | GACTACCGCTGCTCCATTCCA | TCCTCTTCATCACTAATGGGGCTTT |
| Twist (h) | GGCCGGAGACCTAGATGTCATT | CCACGCCCTGTTTCTTTGAAT |
| ZEB1 (h) | GAAAATGAGCAAAACCATGATCCTA | CAGGTGCCTCAGGAAAAATGA |
| ZEB2 (h) | TTCCATTGCTGTGGGCCTT | TTGTGGGAGGGTTACTGTTGG |
| TGF-β1 (r) | AAGAAGTCACCCGCGTGCTA | TGTGTGATGTCTTTGGTTTTGTCA |
| CB2 (h) | TGGCAGCGTGACTATGAC | AAAGAGGAAGGCGATGAA |
| CB2 (r) | ACTGCCTGCTGCGGACATC | GCTGATTGGTCTTCTCACTGAACAC |
| Smad7 (h, r) | CCGCAGCAGTTACCCCATCT | CGAAAGCCTTGATGGAGAAACC |
| IL-1β (r) | CACCTCTCAAGCAGAGCACAG | GGGTTCCATGGTGAAGTCAAC |
| TNF-α (r) | CCAGGTTCTCTTCAAGGGACAA | CTCCTGGTATGAAATGGCAAATC |
| IL-6 (r) | GCCCTTCAGGAACAGCTATG | GCAGTGGCTGTCAACAACA |
| IL-4 (r) | CGTGATGTACCTCCGTGCTT | GTGAGTTCAGACCGCTGACA |
| MCP-1 (r) | CAGGTCTCTGTCACGCTTCTGG | AATGAGTAGCAGCAGGTGAGTGG |
| IL-10(r) | CACTGCTATGTTGCCTGCTCTTAC | GGGTCTGGCTGACTGGGAAG |
| iNOS (r) | GAGTGAGGAGCAGGTTGAGG | CCAAGGTGTTGCCCTTTTT |
| CD86 (r) | GACACCCACGGGATCAATTA | GCCTCCTCTATTTCAGGTTCAC |
| Ym-1 (r) | GATCACCACCCCTATGACCCT | GGGACCAGTTGGTGTAGTAGC |
| CD206 (r) | ACTGCGTGGTGATGAAAGG | TAACCCAGTGGTTGCTCACA |
| Arginase-1 (r) | TTGATGTTGATGGACTGGAC | TCTCTGGCTTATGATTACCTTC |
| GAPDH (h,r) | CATCCTGCACCACCAACTGCTTAG | GCCTGCTTCACCACCTTCTTGATG |

Table S4. Components of the binding free energy (kcal/mol) calculated by MM/GBSA approach.*

| *E*vdw | *E*ele | *G*gb | *G*np | Δ*G*cal |
| --- | --- | --- | --- | --- |
| -76.45 ± 3.07 | -9.99 ± 4.40 | 25.93 ± 4.27 | -9.45 ± 0.23 | -69.96 ± 4.15 |

*The statistical error was estimated based on stabilized 100 ns MD simulation trajectory. 1000 snapshots evenly extracted from the 40-140 ns MD trajectory of complex were used for MM/GBSA calculations.
